# Analysis of Uniformity of Magnetic Field Generated by the Two-Pair Coil System

**DOI:** 10.1007/s00723-012-0427-5

**Published:** 2013-01-08

**Authors:** P. Kędzia, T. Czechowski, M. Baranowski, J. Jurga, E. Szcześniak

**Affiliations:** 1 Laboratory of EPR Tomography Institute of Materials Technology, Faculty of Mechanical Engineering and Management, Poznan University of Technology, Piotrowo 3 St., 60-965 Poznan, Poland; 2 Department of Physics, Adam Mickiewicz University, Umultowska 85, St., 61-614 Poznan, Poland

## Abstract

In this paper we use a simple analysis based on properties of the axial field generated by symmetrical multipoles to reveal all possible distributions of two coaxial pairs of circular windings, which result in systems featuring zero octupole and 32 pole magnetic moments (six-order systems). Homogeneity of magnetic field of selected systems is analyzed. It has been found that one of the derived systems generates homogenous magnetic field whose volume is comparable to that yielded by the eight-order system. The influence of the current distribution and the windings placement on the field homogeneity is considered. The table, graphs and equations given in the paper facilitate the choice of the most appropriate design for a given problem. The systems presented may find applications in low field electron paramagnetic resonance imaging, some functional f-MRI (nuclear magnetic resonance imaging) and bioelectromagnetic experiments requiring the access to the working space from all directions.

## Introduction

Magnetic fields of a specific spatial distribution are used in many areas of physics and technology and analytic approaches to the problem of designing systems, which are capable of generating the desirable fields may be found in the comprehensive surveys papers [1–4] and literature cited therein.

In many applications it is required that the field be highly homogenous over some specified volume. This is of particular importance in magnetic resonance imaging experiments. The systems used for in vivo medical diagnostic studies most often employ solenoidal superconducting electromagnets that are expensive and in certain applications pose disadvantages associated with the limited access to the region of uniform field. In electron paramagnetic resonance imaging (EPRI) [5] and in some functional nuclear magnetic resonance imaging (MRI) experiments [6], electromagnets generating low fields and/or allowing access to the working space from all directions and not just axial are desirable. A classic example of systems satisfying these conditions are air-core assemblies comprising a number of circular or square windings placed co-axially and distributed so that the leading perturbation terms in the field series expansion are eliminated.

In this paper, we consider the system consisting of two coaxial pairs of circular loops with the same radius. The use of properly distributed windings of the same radius makes the radial access to the uniform field possible and does not impose restriction on the axial access, which may sometime occur in systems based on spherical harmonic expansion [4, 10] with outer pair of windings of smaller radius or having a single loop in the mid plane.

We use a simple analysis based on properties of the axial magnetic field with the aim to reveal the possible distributions of windings, that result in systems featuring zero octupole and 32 magnetic moments, i.e., generating the central magnetic field in which the sixth-order term is the first one non-vanishing in the field expansion. The table, formulae, and graphs given in the paper facilitate the choice of design, which is the most suitable for the problem at hand.

The system presented generates extended volume of uniform magnetic field, which can be accessed from all directions. It may be suitable for very-low field MRI and EPRI as well as bioelectromagnetic experiments [7]. The high-field system can be easily shielded by confinement in other with larger radius, which cancels the total dipole moment and results in reduction of the stray field at the expense of a slight decrease of strength of the very homogenous central field.

## System Configuration Analysis

Consider a system of two circular current loops of the same radius $R$, with current $I$ flowing in the same sense in each loop. Let the loops encircle *z*-axis, be located symmetrically with respect to the origin, and separated by the distance $2d$. Using the Biot–Savart law we calculate that the axial field $B_{z}$ is:

1 $$ B_{z} = \frac{{\mu_{0} IR^{2} }}{2}\left[ {\left( {R^{2} + z^{2} - 2zd + d^{2} } \right)^{ - 3/2} + \left( {R^{2} + z^{2} + 2zd + d^{2} } \right)^{ - 3/2} } \right]. $$

Let us now focus the attention on how the axial field behaves outside the coil system and in its central region. To show the behavior of the distant axial $B_{z}$ field, we may expand in the Taylor series the function in square brackets of Eq. (1) in inverse powers of $z$. This yields:

2 $$ B_{z} = c_{k - 1} z^{ - (k + 2)} . $$

It is seen the distant axial field may be viewed as the superposition of magnetic multipoles at the system origin. Due to symmetry of current distribution all coefficients $c_{k - 1}$ for an even $k$ will vanish. This means that in the field expansion there will be no $2^{k}$ poles with $k$ even, just dipole ($k = 1$), octupole ($k = 3$), 32 pole ($k = 5$) and so on.

The first coefficients are found to be:

3a $$ c_{0} = \mu_{o} IR^{2}, $$

3b $$ c_{2} = 3\mu_{0} IR^{2} (4d^{2} - R^{2} )/2, $$

3c $$ c_{4} = 15\mu_{0} IR^{2} \left( {8d^{4} - 12d^{2} R^{2} + R^{4} } \right)/8. $$

The first term in the field expansion $c_{0} z^{ - 3}$ is evidently the axial field due to the dipole at origin ($k = 1$). The second term $c_{2} z^{ - 5}$ is the octupole term ($k = 3$), the third ($k = 5$) $32$ pole term and so on. But we see from Eqs. (2) and (3b) that the octupole term becomes zero if $2d = R$ (the Helmholtz condition). Now as Purcell [9] pointed out, the field of any symmetrical multipole cannot be zero along the symmetry axis unless it is zero off the axis everywhere as well. Consequently, the entire octupole field, not just $B_{z}$ on the axis, vanishes, if $2d = R$. This means that every Helmholtz pair has the zero octupole moment.

By expanding in the Taylor series the function in square brackets of Eq. (1) around the center, we can readily show that the central field generated by the system is:

4 $$ B_{z} = \mathop \sum \limits c_{k - 1} z^{k - 1}, $$

where:

5a $$ c_{0} = \frac{{\mu_{0} IR^{2} }}{{\left( {R^{2} + d^{2} } \right)^{3/2} }}, $$

5b $$ c_{2} = \frac{{3\mu_{0} IR^{2} }}{{2(R^{2} + d^{2} )^{5/2} }} \cdot \frac{{(4d^{2} - R^{2} )}}{{(R^{2} + d^{2} )}}, $$

5c $$ c_{4} = \frac{{15\mu_{0} IR^{2} }}{{8(R^{2} + d^{2} )^{7/2} }} \cdot \frac{{(8d^{4} - 12d^{2} R^{2} + R^{4} )}}{{(R^{2} + d^{2} )}}. $$

Owing to the symmetry of current distribution the axial magnetic field in the central region of the system is an even function of $z$.

But for the Helmholtz condition ($2d = R$) the $c_{2}$ coefficient vanishes, which maximizes the volume and uniformity of the field in the central region of the system. It is possible to make this field still more uniform and to extend its useful volume by using additional pairs of coils, which help to nullify the next coefficient in the field expansion [1].

To eliminate both octupole and 32 pole terms, we consider a system of two coaxial pairs of circular current loops of the same radius $R$, i.e., a four-coil system with number of Amp–turns $(NI)_{1}$ and $(NI)_{2}$, respectively, and current flowing in the same sense in each loop. Let the loops be located symmetrically with respect to the origin of *z*-axis at distances $2d_{1}$ and $2d_{2}$, respectively. From the Biot–Savart law the axial field $B_{z}$ generated by the system is:

6 $$ \begin{aligned} B_{z} & = \frac{{\mu_{0} \left( {NI} \right)_{1} R^{2} }}{2}\left[ {\left( {R^{2} + z^{2} - 2zd_{1} + d_{1}^{2} } \right)^{ - 3/2} + \left( {R^{2} + z^{2} + 2zd_{1} + d_{1}^{2} } \right)^{ - 3/2} } \right] \\ & \quad + \frac{{\mu_{0} \left( {NI} \right)_{2} R^{2} }}{2}\left[ {\left( {R^{2} + z^{2} - 2zd_{2} + d_{2}^{2} } \right)^{ - 3/2} + \left( {R^{2} + z^{2} + 2zd_{2} + d_{2}^{2} } \right)^{ - 3/2} } \right]. \\ \end{aligned} $$

Expanding in the Taylor series the function in square brackets around the center we have:

7 $$ B_{z} = \mathop {\sum \left( {c_{k - 1}^{(1)} + c_{k - 1}^{(2)} } \right)}\limits z^{k - 1}, $$

where $c_{k - 1}^{(1)}$ and $c_{k - 1}^{(2)}$ are the coefficients in the multipole field expansion for respective coil pairs. By analogy to Eqs. (5a, 5b, 5c) we may write:

8a $$ \begin{gathered} c_{0}^{(1)} = \frac{{\mu_{0} (NI)_{1} R^{2} }}{{(R^{2} + d_{1}^{2} )^{3/2} }}, \hfill \\ c_{0}^{(2)} = \frac{{\mu_{0} (NI)_{2} R^{2} }}{{(R^{2} + d_{2}^{2} )^{3/2} }}, \hfill \\ \end{gathered} $$

8b $$ \begin{gathered} c_{2}^{(1)} = \frac{{3\mu_{0} (NI)_{1} R^{2} }}{{2(R^{2} + d_{1}^{2} )^{5/2} }} \cdot \frac{{(4d_{1}^{2} - R^{2} )}}{{(R^{2} + d_{1}^{2} )}}, \hfill \\ c_{2}^{(2)} = \frac{{3\mu_{0} (NI)_{2} R^{2} }}{{2(R^{2} + d_{2}^{2} )^{5/2} }} \cdot \frac{{(4d_{2}^{2} - R^{2} )}}{{(R^{2} + d_{2}^{2} )}}, \hfill \\ \end{gathered} $$

8c $$ \begin{gathered} c_{4}^{(1)} = \frac{{15\mu_{0} (NI)_{1} R^{2} }}{{8(R^{2} + d_{1}^{2} )^{7/2} }} \cdot \frac{{(8d_{1}^{4} - 12d_{1}^{2} R^{2} + R^{4} )}}{{(R^{2} + d_{1}^{2} )}}, \hfill \\ c_{4}^{(2)} = \frac{{15\mu_{0} (NI)_{2} R^{2} }}{{8(R^{2} + d_{2}^{2} )^{7/2} }} \cdot \frac{{(8d_{2}^{4} - 12d_{2}^{2} R^{2} + R^{4} )}}{{(R^{2} + d_{2}^{2} )}}. \hfill \\ \end{gathered}$$

It is seen that both octupole ($k = 3$) and 32 pole ($k = 5$) terms in the field expansion will be zero if:

9a $$ c_{2}^{(1)} + c_{2}^{(2)} = 0, $$

9b $$ c_{4}^{(1)} + c_{4}^{(2)} = 0. $$

The set of Eqs. (9a, 9b) contains three variables $\left\{ {d_{1} ,d_{2} ,(NI)_{1} /(NI)_{2} } \right\}$, one of which can be treated as a parameter. In our calculation the chosen parameter has been the Amp–turn ratio.

The numerical solutions of Eqs. (9a, 9b) may be divided into two families. The first one, which contracts the length of the coils system, and the second one, which extracts the length.

## Numerical Results

**Fig. 1:**
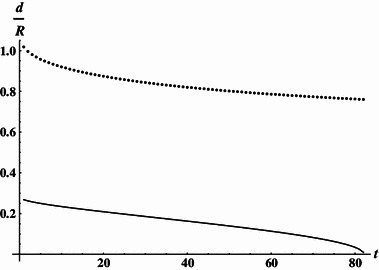
Dimensionless coordinates of coils (contracting geometry). *Solid line* inner coils, *dotted line* outer coils

**Fig. 2:**
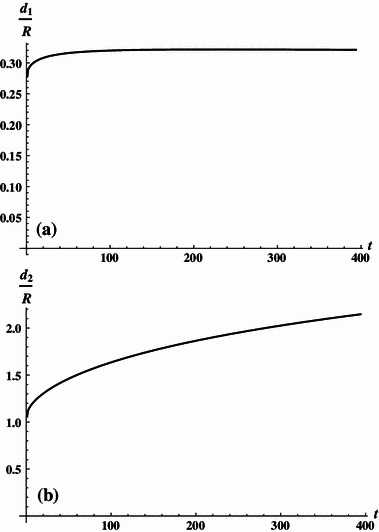
**a** Dimensionless coordinates of inner coils (extracting geometry). **b** Dimensionless coordinates of outer coils (extracting geometry)

Table 1 lists the Amp–turn ratios and the corresponding coil spacing obtained for the family of solutions contracting the coil length. Using the LS approximation we have found that these solutions may be approximated by:

10a $$ \frac{{d_{1} }}{R} = 0.25153 + 0.06065e^{ - t} - 0.00173t - 0.00001t^{2}, $$

10b $$ \frac{{d_{2} }}{R} = 0.96173 - 0.06781e^{ - t} - 0.00466t + 0.22810te^{ - t} + 0.00003t^{2}, $$

10c $$ (NI)_{2} /(NI)_{1} = 2.12 + 0.02t $$

for $1 \le t \le 82$. The obtained solutions are shown graphically in Fig. 1. For the family extracting the length, the solutions may be approximated by:

11a $$ \frac{{d_{1} }}{R} = 0.28115 - 0.00004t + 0.00921\ln t, $$

11b $$ \frac{{d_{2} }}{R} = 1.11207 + 0.01702t + 3.4 \cdot 10^{ - 6} t^{2} - 0.00263t\ln t, $$

11c $$ (NI)_{2} /(NI)_{1} = 2.12 + 0.02t $$

for $1 \le t \le 394$. The latter solutions are shown graphically in Fig. 2a, b. The derived solutions allow easy construction of the four-coil system featuring zero octupole and 32 pole terms.

**Table 1:** Coil spacings and design specifications for contracting systems

*d*_1_/*R*	*d*_2_/*R*	(*NI*)_2_/(*NI*)_1_	*B*_*z*_ (mG)	*d*_1_/*R*	*d*_2_/*R*	(*NI*)_2_/(*NI*)_1_	*B*_*z*_ (mG)	*d*_1_/*R*	*d*_2_/*R*	(*NI*)_2_/(*NI*)_1_	*B*_*z*_ (mG)
0.26825	1.01837	2.l4	82.2879	0.19375	0.85304	2.65	106.22	0.13422	0.79843	3.l6	124.74
0.26489	1.00574	2.15	83.2848	0.1926	0.85159	2.66	106.598	0.13297	0.79763	3.17	125.093
0.26213	1.12189	2.16	77.4822	0.19145	0.85017	2.67	106.975	0.13171	0.79684	3.18	125.446
0.2597	0.98779	2.17	84.8545	0.19031	0.84876	2.68	107.352	0.13044	0.79605	3.19	125.799
0.25748	0.98065	2.18	85.5344	0.18916	0.84738	2.69	107.727	0.12917	0.79527	3.2	126.151
0.25541	0.97425	2.19	86.1717	0.18801	0.84602	2.7	108.102	0.1279	0.7945	3.21	126.503
0.25347	0.96843	2.2	86.7767	0.18687	0.84468	2.71	108.476	0.12661	0.79374	3.22	126.855
0.25161	0.96306	2.21	87.356	0.18573	0.84335	2.72	108.849	0.12532	0.79298	3.23	127.207
0.24983	0.95808	2.22	87.9144	0.18459	0.84204	2.73	109.221	0.12402	0.79223	3.24	127.558
0.24811	0.95341	2.23	88.4553	0.18345	0.84076	2.74	109.593	0.12271	0.79148	3.25	127.909
0.24645	0.94901	2.24	88.9814	0.1823	0.83949	2.75	109.963	0.12139	0.79074	3.26	128.26
0.24483	0.94485	2.25	89.4946	0.18116	0.83823	2.76	110.334	0.12006	0.79001	3.27	128.611
0.24325	0.9409	2.26	89.9967	0.18002	0.837	2.77	110.703	0.11873	0.78928	3.28	128.962
0.24172	0.93714	2.27	90.489	0.17888	0.83578	2.78	111.072	0.11738	0.78856	3.29	129.312
0.24021	0.93354	2.28	90.9724	0.17774	0.83457	2.79	111.44	0.11602	0.78785	3.3	129.662
0.23873	0.93008	2.29	91.448	0.1766	0.83338	2.8	111.808	0.11466	0.78714	3.31	130.013
0.23727	0.92677	2.3	91.9165	0.17546	0.83221	2.81	112.175	0.11328	0.78643	3.32	130.362
0.23584	0.92358	2.31	92.3786	0.17432	0.83105	2.82	112.541	0.11189	0.78574	3.33	130.712
0.23444	0.9205	2.32	92.8348	0.17318	0.8299	2.83	112.907	0.11049	0.78504	3.34	131.061
0.23305	0.91753	2.33	93.2856	0.17204	0.82877	2.84	113.272	0.10907	0.78436	3.35	131.411
0.23168	0.91465	2.34	93.7314	0.17089	0.82765	2.85	113.637	0.10765	0.78368	3.36	131.76
0.23032	0.91186	2.35	94.1727	0.16975	0.82655	2.86	114.001	0.10621	0.783	3.37	132.109
0.22898	0.90916	2.36	94.6098	0.1686	0.82545	2.87	114.365	0.10475	0.78233	3.38	132.458
0.22766	0.90654	2.37	95.0429	0.16745	0.82438	2.88	114.728	0.10328	0.78167	3.39	132.806
0.22634	0.90399	2.38	95.4723	0.16631	0.82331	2.89	115.091	0.1018	0.78101	3.4	133.155
0.22504	0.90151	2.39	95.8983	0.16516	0.82226	2.9	115.453	0.1003	0.78035	3.41	133.503
0.22376	0.8991	2.4	96.3211	0.164	0.82121	2.91	115.815	0.09878	0.7797	3.42	133.851
0.22248	0.89675	2.41	96.7409	0.16285	0.82018	2.92	116.176	0.09724	0.77906	3.43	134.199
0.22121	0.89446	2.42	97.1578	0.16169	0.81916	2.93	116.537	0.09569	0.77842	3.44	134.547
0.21995	0.89222	2.43	97.572	0.16054	0.81816	2.94	116.898	0.09411	0.77779	3.45	134.895
0.2187	0.89003	2.44	97.9838	0.15938	0.81716	2.95	117.258	0.09251	0.77716	3.46	135.242
0.21746	0.8879	2.45	98.3931	0.15821	0.81617	2.96	117.618	0.0909	0.77653	3.47	135.59
0.21623	0.88581	2.46	98.8002	0.15705	0.8152	2.97	117.977	0.08926	0.77591	3.48	135.937
0.215	0.88377	2.47	99.2051	0.15588	0.81423	2.98	118.336	0.08759	0.7753	3.49	136.284
0.21378	0.88177	2.48	99.608	0.15471	0.81328	2.99	118.694	0.0859	0.77468	3.5	136.631
0.21256	0.87982	2.49	100.009	0.15354	0.81233	3.	119.053	0.08418	0.77408	3.51	136.978
0.21136	0.8779	2.5	100.408	0.15236	0.8114	3.01	119.411	0.08243	0.77348	3.52	137.324
0.21015	0.87603	2.51	100.805	0.15118	0.81047	3.02	119.768	0.08065	0.77288	3.53	137.671
0.20896	0.87418	2.52	101.201	0.15	0.80956	3.03	120.125	0.07883	0.77228	3.54	138.017
0.20777	0.87238	2.53	101.595	0.14881	0.80865	3.04	120.482	0.07698	0.77169	3.55	138.364
0.20658	0.87061	2.54	101.988	0.14762	0.80775	3.05	120.838	0.07509	0.77111	3.56	138.71
0.2054	0.86887	2.55	102.379	0.14642	0.80687	3.06	121.195	0.07316	0.77053	3.57	139.056
0.20422	0.86716	2.56	102.768	0.14522	0.80599	3.07	121.55	0.07118	0.76995	3.58	139.402
0.20304	0.86548	2.57	103.156	0.14402	0.80511	3.08	121.906	0.06915	0.76938	3.59	139.748
0.20187	0.86383	2.58	103.543	0.14281	0.80425	3.09	122.261	0.06707	0.76881	3.6	140.094
0.2007	0.86221	2.59	103.929	0.1416	0.8034	3.1	122.616	0.06493	0.76824	3.61	140.439
0.19954	0.86062	2.6	104.314	0.14038	0.80255	3.11	122.971	0.06272	0.76768	3.62	140.785
0.19838	0.85906	2.61	104.697	0.13916	0.80171	3.12	123.325	0.06044	0.76713	3.63	141.13
0.19722	0.85751	2.62	105.079	0.13793	0.80088	3.13	123.679	0.05808	0.76657	3.64	141.475
0.19606	0.856	2.63	105.46	0.1367	0.80006	3.14	124.033	0.05563	0.76602	3.65	141.82
0.1949	0.85451	2.64	105.841	0.13546	0.79924	3.15	124.387	0.05307	0.76548	3.66	142.165

For the family of solutions contracting the coils length one set of the derived variables corresponds well to that obtained previously by means of the Bessel functions formalism by Lee-Whiting [8]. As it is seen from Table 1, for $\left( {NI} \right)_{2} /\left( {NI} \right)_{1} = 2.26$ we have calculated $d_{1} /R = 0.24325$ and $d_{2} /R = 0.9409$ in good agreement with the respective variables: 2.2604, 0.24319 and 0.94073 given in Ref. [7].

## Analysis of the Magnetic Field Uniformity

**Fig. 3:**
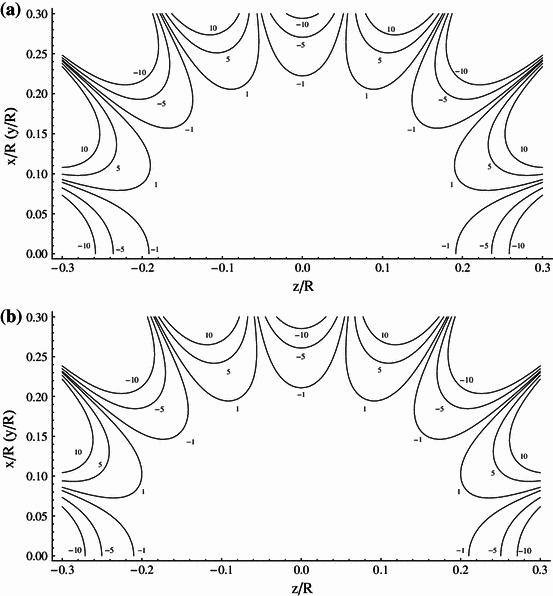
**a** Contours of the constant magnetic field plotted at 1, 5, 10 ppm intervals (ideal system proposed by Lee-Whiting). **b** Contours of the constant magnetic field plotted at 1, 5, 10 ppm intervals (ideal (*NI*)_2_/(*NI*)_1_ = 9/4 contracting system)

Hereafter, we consider in more detail the $\left( {NI} \right)_{2} /\left( {NI} \right)_{1} = 9/4$ system, which has the potential of being power supply in series. For this Amp–turn ratio the windings are positioned at $d_{1} /R = 0.24483$ and $d_{2} /R = 0.94485$. To compare performance of the system with that of Lee-Whiting [8], we have analyzed the spatial distribution of the magnetic field generated by both systems. The evaluation of the field homogeneity involved the use of the Biot–Savart relation applied to small segments of windings. Results of analysis are presented in Fig. 3a, b, which show contours of constant magnetic field relative to the field at center (field error contours) defined as (*B*_given point_ − *B*_center_)/*B*_center_ plotted at ±1, ±5, and ±10 ppm intervals for the ideal eight-order Lee-Whiting design and our 9/4 system, respectively. It is seen that that the field error contours of both systems compare excellently and the volume of the homogenous magnetic field is similar.

**Fig. 4:**
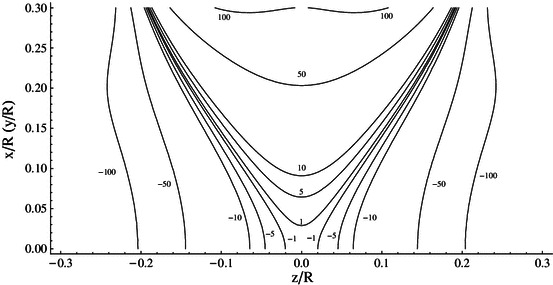
Contours of the constant magnetic field plotted at 1, 5, 10, 50, 100 ppm intervals (system of Lee-Whiting with 9/4 Amp–turn ratio)

In Ref. [11] the Amp–turn ratio of 2.2604 given by Lee-Whiting is approximated by 9/4 (2.25) and the windings placements are left unchanged. Such an approximation expressing the numerator and denominator of the Amp–turn ratio by integers allows the design of systems having coils connected in series and fed by one common source, which is most preferable in practical applications. Unfortunately, as we show in Fig. 4 this small change of only one parameter has a significant impact on the system performance, leading to a very substantial reduction of the generated field homogeneity.

**Fig. 5:**
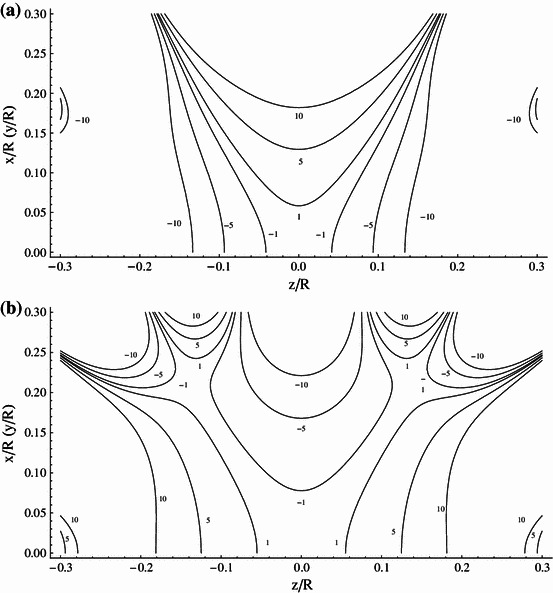
**a** Contours of the constant magnetic field plotted at 1, 5, 10 ppm intervals (ideal Lee-Whiting system with 3 significant digits for wires distribution). **b** Contours of the constant magnetic field plotted at 1, 5, 10 ppm intervals [(*NI*)_2_/(*NI*)_1_ = 9/4 contracting system with 3 significant digits for wires distribution]

In Table 1 we specify the coil’s position with an accuracy of 5 significant digits. With the same accuracy the location of windings is given in the Lee-Whiting paper. Such a precision, however, would be difficult to achieve when constructing the real system. To evaluate the influence of the location of coils on the field homogeneity, we have calculated the field error contours of both systems assuming the coils placement specified with the accuracy of 3 significant digits, i.e., we have assumed $d_{1} /R = 0.243$ and $d_{2} /R = 0.941$ for the Lee-Whiting system and $d_{1} /R = 0.245$ and $d_{2} /R = 0.945$ for the system presented in this paper. The results of calculation are plotted in Fig. 5a, b for the Lee-Whiting arrangement and our 9/4 system, respectively. When comparing pair of Figs. 3a, 5a with the pair 3b and 5b, we see that the 9/4 restricted system generates the greater volume of homogenous magnetic field and is less sensitive to the precision of the location of coils.

**Fig. 6:**
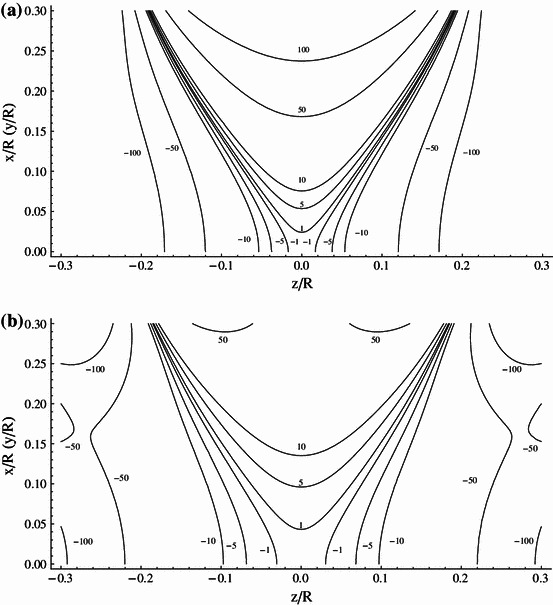
**a** Contours of the constant magnetic field plotted at 1, 5, 10, 50, 100 ppm intervals (real Lee-Whiting system). **b** Contours of the constant magnetic field plotted at 1, 5, 10, 50, 100 ppm intervals [real (*NI*)_2_/(*NI*)_1_ = 9/4 contracting system]

The real assemblies with coils connected in series consist of many turns of wire fed with equal current and their Amp–turn ratio (*NI*)_2_/(*NI*)_1_ depends solely on the number of windings of the outer and inner coils. As an example of such assemblies, we have analyzed the arrangements comprising nine circular current loops in the outer coils and four loops in the inner coils. The loops are deposited side by side around *z* coordinates corresponding to the Lee-Whiting and our 9/4 contracted designs. The expected distributions of the magnetic field generated by the two designs are shown in Fig. 6a, b, respectively. It is seen that the setup suitable for the serial power supply based on our 9/4 system produces much greater volume of the homogeneous magnetic field than that using the Lee-Whiting coordinates.

The setup based on the 9/4 contracted system having the unit radius (*R* = 1 m) and fed with the current 1 A generates magnetic field whose value in the center is 89.5 × 10^−7^ T (89.5 mG). The field homogeneous to 50 ppm, which meets, e.g., EPRI requirements, extends axially to 0.22*R* and radially to 0.3*R* from the center. In the case of setup that uses the Lee-Whiting coordinates, the field value is 89.9 × 10^−7^ T and the field homogeneous to 50 ppm extends axially to 0.11*R* and radially to 0.17*R* from the center.

The magnetic field homogeneity has been analyzed for all systems listed in Table 1. The maximum volume of the homogeneous field is generated by the $\left( {NI} \right)_{2} /\left( {NI} \right)_{1} = 2.26$ system. When the Amp–turn ratio changes the volume of the uniform magnetic field slowly decreases. For the last system listed in Table 1 the field homogeneous to 1 ppm extends axially to 0.11*R* and radially to 0.13*R*. The value of field is 142 × 10^−7^ T.

## Shielding of the System

The distant (stray) field of the two-pair coil system may be easily shielded. To show the behavior of the distant axial $$ B_{z}, $$ we may expand Eq. (6) in inverse powers of $$ z $$. This yields:

12 $$ B_{z} = \mathop \sum \limits_{k} (c_{k - 1}^{(1)} + c_{k - 1}^{(2)} )z^{ - (k + 2)} . $$

The first three turns in the expansion are readily found to be those for $k = 1$, $k = 3$ and $k = 5$ with coefficients:

13a $$ c_{0} = \mu_{o} [(NI)_{1} + (NI)_{2} ]R^{2}, $$

13b $$ c_{2} = 3\mu_{0} (NI)_{1} R^{2} (4d_{1}^{2} - R^{2} )/2 + 3\mu_{0} (NI)_{2} R^{2} (4d_{2}^{2} - R^{2} )/2, $$

13c $$ \begin{gathered} c_{4} = 15\mu_{0} (NI)_{1} R^{2} \left( {8d_{1}^{4} - 12d_{1}^{2} R^{2} + R^{4} } \right)/8 \hfill \\ + 15\mu_{0} (NI)_{2} R^{2} \left( {8d_{2}^{4} - 12d_{2}^{2} R^{2} + R^{4} } \right)/8. \hfill \\ \end{gathered} $$

So, we see that the external (stray) field of the system is dominated by the dipolar term and decreases as $z^{ - 3}$. However, in some applications, it is required that the central field be very homogenous and at the same time the stray field be falling off rapidly. Could we somehow suppress the dipolar term of the four-coil system? Yes, and it is rather easy to do. We can surround the system with another one of larger radius with opposed currents arranged so dipolar moments of the systems given by Eqs. (13a, 13b, 13c) cancel.

**Fig. 7:**
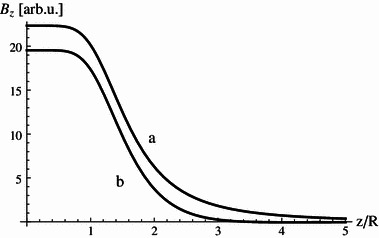
Axial magnetic field generated by **a** four-coil system, **b** nested four-coil system

For example, if the outer system has twice the radius of the inner one, we need the current flowing in it to be four times smaller than that in the inner system to achieve cancellation. Adding the outer system cancels the total dipole moment not effecting the homogeneity of the central field, but slightly reducing the field value. For the case considered it amounts to 12.5 %.The behavior of the stray field is shown in Fig. 7, it does fall off faster to zero when the whole nested system is energized compared with the inner four-coil system alone.

It is clear that the confinement provides a simple system with the reduced stray field, which in some circumstances may prove to be useful.

## Conclusions

The possible distributions of two coaxial pairs of circular windings resulting in systems featuring zero octupole and 32 pole moments are given. Analysis given in the paper shows that one of the derived system, which has the Amp–turn ratio of 9/4, generates homogeneous magnetic field whose volume compares excellently with that of the eight-order Lee-Whiting design, but is less sensitive to the impact of current distribution and the precision of windings location. Moreover, the system may be more easily adopted for the serial power supply. The ideal 9/4 system of the unit radius fed with 1 A current generates the magnetic field of 89.5 mG, which is about 10 times stronger than that yielded by the Helmholtz pair. The field is proportional to the number of Amp–turns and scales with *R* ^−1^, hence systems of smaller radius and/or carrying higher currents will generate higher magnetic fields. Yet, to avoid heating problems they will require thermal and current stabilization. This refers particularly to long-time experiments. Based on the design presented, we plan to build 90 G magnet for micro-EPRI experiments, which should shed some light on the problems associated with practical implementation of the system. Finally, we would like to add that the high-field version of the system can be easily shielded by confinement of the system inside another one with bigger radius. This exerts no significant influence on the center field, but reduces greatly the stray field outside.
